# Editorial: Hospitalization and Parkinson's disease: safety, quality and outcomes

**DOI:** 10.3389/fnagi.2024.1398947

**Published:** 2024-04-04

**Authors:** Hooman Azmi, Benjamin L. Walter, Annie Brooks, Irene Hegeman Richard, Katherine Amodeo, Michael S. Okun

**Affiliations:** ^1^Department of Neurosurgery, Hackensack University Medical Center, Hackensack, NJ, United States; ^2^Hackensack Meridian School of Medicine, Nutley, NJ, United States; ^3^Cleveland Clinic, Cleveland, OH, United States; ^4^Parkinson's Foundation, New York, NY, United States; ^5^Department of Neurology, University of Rochester Medical Center, Rochester, NY, United States; ^6^Westchester Medical Center Health Network, Valhalla, NY, United States; ^7^Department of Neurology, Norman Fixel Institute for Neurological Diseases, University of Florida, Gainesville, FL, United States

**Keywords:** Parkinson's disease, hospitalization, safety, risks, quality

## Introduction

For people with Parkinson's (PWP), hospital admissions can be perilous. Nearly 300,000 PWP are admitted to the hospital each year in the US. Following admission, they are at an increased risk of complications that may lengthen their stay and increase the risks of both morbidity and mortality. These preventable hospital-occurring complications occur as a result of many factors.

The majority of PWP enter the hospital for non-PD related issues and are placed in alternative units rather than the neurology floor. Treatment teams may not be cognizant of a patient's PD diagnosis or may alternatively be unfamiliar with special considerations for hospital safety for PWP.

The Parkinson's Foundation Hospital Care Recommendations were recently created as a step toward eliminating preventable harm for PWP in the hospital. To advance this work, we as a field realized that there is a need to demonstrate the prevalence of challenges with both medication administration and overall management of the PWP in the hospital. To achieve meaningful data on patient outcomes and to realize cost savings, we need hospital systems to engage with quality champions and to harness the full potential of information technology and electronic heath records (EHR).

Our Topic series “*Hospitalization and Parkinson's disease: safety, quality and outcomes*” was intended to encourage the discourse surrounding this issue and to further expand the knowledge base. This series includes thirteen publications written from varied perspectives, all centered on the hospitalization of PWP. The articles draw attention to the risks that PWP face in the hospital by providing a clearer idea of the magnitude of existing gaps in care, exploring of the impact of these gaps on both clinical and economic outcomes, and identifying best practices. This editorial is focused on three key themes: (1) understanding risk and outcomes, (2) improving hospital care, and (3) exploring hospitalization through community perspectives.

## Understanding risk and outcomes for PWP

For over a decade, the Parkinson's Foundation has worked diligently to shed light on hospital safety gaps and encourage the development of solutions. Significant gains were made through an initiative led by Michael Okun through collaboration among the Parkinson's Foundation Global Care Network resulting in publications of dozens of articles identifying the risks to PWP (Magdalinou et al., 2007; Buetow et al., 2010; Derry et al., 2010; Wood et al., 2010; Aminoff et al., 2011; Chou et al., 2011; Delea et al., 2011; Gerlach et al., 2011, 2013; Hou et al., 2012; Wawruch et al., 2012; Anderson and Fagerlund, 2013; Fagerlund et al., 2013; Hassan et al., 2013; Ahlskog, 2014; Cohen and Smetzer, 2015; Crispo et al., 2016; Shahgholi et al., 2017). Their research also led to the creation of the Parkinson's Foundation hospital safety kits, which have been distributed to over 150,000 PWP. As community awareness increased, investigators better defined the safety gaps and demonstrated the effect on PWP (Gerlach et al., 2012; Oguh and Videnovic, 2012; Martinez-Ramirez et al., 2015).

In this series, we offer a more comprehensive and detailed effort at defining the problem. One review analyzes 35,457 admissions for PWP and explores the complications and outcomes. This article showed an increased risk of delirium and aspiration pneumonia, however interestingly, neither falls nor UTI were cited as a big challenge (George et al.). A smaller study in the series found that while patients with parkinsonism and psychosis had a higher rate of hospitalization, the duration of hospitalization was consistent whether psychosis was active, resolved, or not present (Piat et al.).

Two articles explored the relationship between the end-of-life period and hospitalization for PWP. A large study of Medicare data observed that over 60% of decedents with PD were hospitalized at least once in their last 6 months of life. This data was compared to 18% of non-decedents (Aamodt et al.). Another study also examined the experiences of hospitalized PWP during the end-of-life period, finding that the majority did not receive palliative care consultations. Lack of consultations was correlated with higher healthcare resource utilization, and the lack of provision of this service was inconsistent with patient and family expectations (Bhansali et al.).

## Exploring community perspectives

Though presenting hospital care risk and outcome data is essential, it presents an incomplete story. Several articles in the series focused on addressing another essential component less represented in the literature: the community perspective. One qualitative study captured the nuances of complex emotional and physical shortfalls in care as expressed by PWP and their care partners. Aligning directly with several of the Parkinson's Foundation Hospital Care Recommendations, PWP expressed the expectation to be recognized as patients with unique needs, especially needs related to mobility and their Parkinson's medication management. PWP and care partners felt that Parkinson's related challenges should be managed collaboratively (Shurer et al.).

Another study in the Research Topic outlined the perception of safety among PWPs receiving care and identified two relevant themes: (1) the importance of access to interdisciplinary care from inpatient clinicians and (2) the necessity for a care team with an adequate understanding of PD (Pedrosa et al.).

Another article reviewed a case example, as recalled by a Parkinson's care partner. This perspective article explored the role care partners could play as advocates and how hospital staff could utilize care partners as active participants in care, a role that half of care partners “hoped to fill”. Communication and a willingness to see the care partner as an expert were identified as primary factors for improving the hospitalization experience. Additionally, this may also minimize risks for aspiration pneumonia (Brooks).

## Improving care

Finally, our Research Topic focused on improving care for PWP. As the literature has evolved from early efforts focused mostly on identifying and better defining the hospitalization challenge, more recent efforts have focused on how processes can impact hospital safety gaps and improve patient care (Skelly et al., 2014; Azmi et al., 2019; Hobson et al., 2019; Nance et al., 2020).

This hospitalization issue expands on tangible efforts to improve care. One article reported on improvement across all chosen performance measures by utilizing the Nurse Professional Development Model. The Model included implementation of onboarding policies, multimodal education, competency management tools (such as time-critical alerts in the Medication Administration Record), development of a “nurse champion” role, collaborative interdisciplinary partnerships, and the development of a process for inquiry into the effectiveness of interventions (Bobek et al.). Another article in the series reviewed how the same center was able to impact care through EHR-based interventions. Using this method, when PD medications were placed within a custom schedule (~14,000 orders), rather than with default options (~17,000), medications were 1.4 times more likely to be administered within 15-min of the scheduled time (Azmi et al.), in alignment with the Parkinson's Foundation Hospital Care Recommendations.

The impact of using a Best Practice Alert (BPA)—another EHR-based intervention—was highlighted in two articles. In both experiences the method successfully reduced the receipt of contraindicated medications in PWP. In one article, administrations decreased by nearly half from 16 to 8.8% (Chunga et al.). Another article showed similar results in the first 3 months of a program, with less significant impact, though still improved, when followed to 1 year (Goldin et al.).

More comprehensive EHR-solutions with pointers for healthcare system leaders were highlighted in two additional articles. One highlighted default features in the Epic “Foundation System,” developed and implemented at medical centers across the country (Wu et al.). Another highlighted a series of recommended policies and tools focused on inpatient pharmacy departments (Yu et al.). Together, these articles provided specific recommendations on how institutions can (1) improve the administration of time-critical PD medications, (2) reduce omissions and substitutions of unavailable medications, and (3) reduce the administration of contraindicated medications.

## Conclusion

We believe that the optimal approach to drive improvements in hospital-based care for PWP will be a systematic and nation-wide quality improvement effort. A cornerstone for this effort is the creation, formalization, and meaningful adoption of clinical guidelines driven by new data and innovative methods. Another cornerstone is the formation of a national community of practice to share learning and accelerate adoption of effective interventions, such as the efforts initiated by the Parkinson's Foundation through their national learning collaborative which includes twenty health systems. Delineating the financial impact of costs incurred to ensure compliance and cost savings associated with harm reduction are also critically important. This compilation of articles provides the data and perspectives we will need to continue on the journey toward improvement.

## Author contributions

HA: Writing – original draft, Writing – review & editing. BW: Writing – review & editing. AB: Writing – original draft, Writing – review & editing. IR: Writing – review & editing. KA: Writing – review & editing. MO: Writing – review & editing.
